# The Service of Medicine to Civilization

**Published:** 1914-08-15

**Authors:** 


### THE SERVICE OF MEDICINE TO CIVILIZATION.

Extracts from an address by Victor C. Vaughan, M.D., President of the American Medical Association June 1914.

In ancient times, civilization was born, grew for a few generations and fell into decay. In all instances it was local and covered only small areas. Its habitations were oases in the world-wide desert of ignorance and superstition, and after an ephemeral existence all were buried in the sand. Relatively small bodies of men occupying salubrious regions developed the elements of science and for a few centuries flourished. Their superior knowledge gave them dominion over their less fortunate neighbors, who were converted into slaves. Conquest brought disease and the local civilizations were obliterated by contagion. History is replete with instances in which triumphant heroes have brought to their rejoicing countries with their prisoners of war invisible and intangible agents of death, which have ultimately vanquished the victors.

The Egyptians of the Pharaohs drained the land, built aqueducts, disposed of their dead hygienically, reared temples and cities, maintained law and order, developed the elements of literature and science and devised and employed simple machinery. In speaking of the ancient Egyptians, Diodorus says: "The whole manner of life was so evenly ordered that it would appear as though it had been arranged by a learned physician, rather than by a lawgiver." Herodotus declared ancient Egypt the healthiest of countries, but filled with physicians of whom "one treats only the diseases of the eye, another those of the head, the teeth, the abdomen or the internal organs." Writing of a later time, Gibbon said: "Ethiopia and Egypt have been stigmatized in all ages as the original source and seminary of the plague." It is evident that in the time of its great civilization Egypt was salubrious; coincident with the decline in the learning and wisdom of its people, it was visited and desolated by pestilence. That Egypt had lost its salubrity as early as the period of the exodus of the Israelites is shown by many passages in the Bible in which the chosen people are threatened with the disease of Egypt if they neglect or violate the laws. Moses, "learned in all the wisdom of the Egyptians," codified his sanitary rules and regulations in the form of religious rites and ceremonies and thus secured their observance among the faithful even down to the present time.

The claim has been advanced that the infectious diseases have benefited the race by the destruction of the unfit. This idea I have combated most vigorously since our study of typhoid fever in the Army in 1898. My colleagues and I found that out of 9,481 soldiers who had previously been on the sick report and could not be regarded as possessing standard health, 648, or 6.8 per cent., contracted typhoid fever; whereas, out of 46,384 men who had no preceding illness, 7,197, or 15.3 per cent., developed typhoid fever. More than 90 per cent. of the men who developed typhoid had no preceding intestinal disorder. Under ordinary conditions the strong, busy man, especially the one whose activities demand wide excursions from his home, is more likely to become infected than the one whose sphere of action is more limited on account of infirmity. The reason for this is too obvious to need statement, and it follows that more men than women and more adults than children have typhoid fever. Moreover, the case mortality is greater among the strong, because death in the infectious diseases is often due to the rapidity with which the invading organism is broken up by the secretions of the body cells and the protein poison made effective. From this I have concluded that contagion, like war, destroys the very flower of the race. This view is sustained by the historians of the pestilences of former times.

Thucydides in his description of the plague at Athens says: "Moreover, no constitution, whether in respect of strength or weakness, was found able to cope with it; nay it swept away all alike, even those attended to with the most careful management."

Procopius in his account of the Justinian epidemic states that youth was the most perilous season, and females less susceptible than males.

Hurty, nearly a century ago, wrote: "A fever which consigns thousands to the grave, consigns tens of thousands to a worse fate—to hopeless poverty, for fever spares the children and cuts off the parents, leaving the wretched offspring to fill the future ranks of prostitution, mendicancy and crime."

The middle ages were indeed dark physically, intellectually and morally. Here and there, now and then, some man of genious towered above the general low level of his contemporaries and not infrequently he paid dearly for his audacity. For some centuries the Arab, especially in Spain, stood out alone as the torch-bearer of science, and he, when driven back into the insalubrity of Northern Africa, lapsed into barbarism. Neuburger writes:

Fortunately the fate of medieval medicine was not dependent on Byzantium alone. An admirable illustration of the doctrine of conservation of energy is afforded by the fact that, with the decline of intellectual energy at home, a contemporaneous development of Greek medicine took place abroad, which, if at times misguided, was yet full of vitality, whilst the medical art of the newly arisen world of Islam reached a height unsurpassed during the middle ages.

In the greater part of Europe, ignorance and disease held full sway. In the midst of great calamities "the will-o-the-wisp of superstition is an irresistible attraction and offers the only ray of hope." Strong men, neglectful of their earthly duties, betook themselves to secluded places and lost themselves in dreams of a heavenly paradise. Mysticism, fanaticism and superstition dominated all conditions of men. Rulers, illiterate, immoral and even incestuous, occupied palaces while the masses died of starvation. The history of the time is a record of diseased, degenerated, demented man. There can be no doubt that disease has overthrown civilizations in the past, and there is no surety that it may not do so again. The recent outbreak of the plague in Manchuria and its more recent appearance in Cuba are not without their warnings. It remains to be seen if those who control our government have the intelligence necessary to protect our country against the invasion of pestilence. The failure to provide for camp sanitation in 1898, the behavior of California officials on the finding of plague in San Francisco and the general indifference of national and state authorities towards the eradication of disease discourage the hope that intelligent patriotism is widely distributed among us. As a contemporary of Mr. Dowie and Mrs. Eddy and as a citizen of a country in which the osteopath and chiropractic flourish, I feel some embarrassment in speaking of the fanaticism and ignorance of the dark ages.

A study of epidemics shows that in the presence of widespread contagion mankind in the mass tends to revert to the barbaric state. This is the unvarying testimony of all authorities, medical and lay, secular and religious, who have made the records.

It is not necessary to turn to history for examples of the degrading effects of disease on man. We see it to-day in the physical inferiority, intellectual weakness and moral irresponsibility of those people who are still under the domination of malaria and kindred diseases. My illustrious predecessor in this office, Dr. Gorgas, has demonstrated what scientific medicine may accomplish in these pestilential regions, and it is within reason to look forward to the time when the tropics may supply choice locations for civilized man. In like manner the valleys of the Tigris and Euphrates are being reclaimed and Babylon and Nineveh may again become seats of learning and culture. The modern sanitarian is quite competent to rebuild the home in which the cradle of civilization was rocked.

Hoffman states that had the death-rate for tuberculosis in 1901 continued there would have been 200,000 more deaths from this cause from that date to 1911 than actually did occur, or the actual saving of lives from death by tuberculosis accomplished in that decennium averaged 20,000 per year. A battle in which 20,000 are slain stirs the world at the time and fills pages of history later. Preventive medicine measures its successes by the number of lives saved, and 20,000 a year preserved from death from one disease is no small triumph. In the last century the average of human life has been increased fifteen years and this increase could be duplicated in the next twenty years if the facts we now possess were effectively employed.

Hoffman further states that the addition to the material wealth of this country secured by the reduction of deaths from tuberculosis within ten years amounts approximately to 6,200,000 years of human life, covering its most productive period. Medicine discovered the facts which have made this great work possible and has directed their application.

With evidence of this kind before them, will our lawmakers listen to those who demand recognition as practitioners of medicine without proper qualification?

The further developments of medicine, both curative and preventive, depend on scientific investigations. The public is the beneficiary and should in every way encourage medical research. By the application of discoveries already made, the burden of disease has been lightened, sickness has become less frequent and less prolonged, a greater degree of health has been secured, the efficiency of the individual and of the nation has been increased and life has been prolonged and made more enjoyable. The federal government and the states should sustain and promote scientific research. That government is the best which secures for its citizens the greatest freedom from disease, the highest degree of health and the longest life, and that people which most fully secures the enjoyment of these blessings will dominate the world.

Medicine consists of the application of scientific discovery to the prevention and cure of disease. All else which may go under the name of medicine is sham and fraud. Without advancement in the physical, chemical and biologic sciences there can be no progressive movement in medicine. Scientific knowledge is gained only by observation and experiment. Before the time of Jenner, we are told by the historian, it was unusual to meet in London one whose face was not marked by small-pox. There was a popular belief that one who had cow-pox was immune to small-pox. Jenner put this belief to a scientific test and the result was the discovery of vaccination, and this secured the abolition of this disfigurement and a marked reduction in mortality.

In 1849, a village doctor, with a crude microscope, studied the blood of animals sick with anthrax and compared it with that of healthy ones. He discovered the anthrax bacillus. This work was extended by Davaine, Pasteur, Koch and others, and from this the science of bacteriology has been developed. The particulate causes of many infectious diseases have been recognized, isolated and their effects on animals demonstrated. Many of the mysteries of contagion have been revealed and the conditions of the transmisson of disease made known. The fundamental principles of preventive medicine have been developed into a science which is to-day the most potent factor in the progress of civilization.

Findlay suspected a certain mosquito to be the carrier of the virus of yellow fever. Reed and his co-workers demonstrated the truth of this theory and the work of Gorgas has freed Havana from the pestilence and the construction of the Panama Canal is an accomplished fact.

The experiments of Villemin demonstrated the contagious nature of tuberculosis, long suspected and frequently denied. The diligent research of Koch resulted in the recognition and isolation of the causative agent, and since this discovery the mortality of the Great White Plague in Europe and the United States has been diminished more than half, and it is within the range of sanity to look forward to the time, when the former "Captain of the hosts of death" will be known only by the fearful records he once made in the history of man's struggle to be relieved from the heavy tribute paid to infection.

We boast of a great civilization, but this is justified only within limits. Science more nearly dominates the world than at any time in the past. Learning permeates the masses more deeply, but credulity and ignorance are widely prevalent. In this country of nearly one hundred millions, there are thousands whose greed impedes the progress of the whole, tens of thousands whose ignorance retards their own growth, and other thousands who live by crime and procreate their kind to feed on generations to come. We have our schools, colleges and universities, while our almshouses, insane asylums and penal institutions are full. In our cities we see the palatial homes of the ultra rich, the splendid temples of trade and commerce, the slums of want and poverty and the homes, both rich and squalid, of vice and crime.

No nation in this condition can be given a clean bill of health. Our hill-tops are illuminated by the light of knowledge, but our valleys are covered by the clouds of ignorance. We have not emerged from the shadows of the dark ages. The historian of the future will have no difficulty in convincing his readers that those who lived at the beginning of the twentieth century were but slightly removed from barbarism, as he will tell that the school, saloon and house of prostitution flourished in close proximity; that the capitalist worked his employees under conditions which precluded soundness of body; that the labor union man dynamited buildings; that whilst we sent missionaries to convert the Moslem and the Buddhist ten thousand murders were committed annually in our midst, and that a large percentage of our mortality was due to preventable disease.

Evidently there is much to be done before we pass out from the shadows of ignorance into the full light of knowledge. In this great work for the betterment of the race the medical profession has important duties to perform. I do not mean to imply that the uplift of mankind devolves wholly on the medical man. The burdens are too many and too diversified, the ascent too steep and the pathways too rough for one profession to hope to reach unaided the high plateau we seek. Moreover, other callings have no right, and should have no desire, to shirk the moral responsibilities, which rest alike on all. But in past ages, medical men have been the chief torch-bearers of science, the only light in which man can safely walk, and we must keep and transmit to our successors this trust and honor.

In so great a work as the eradication of preventable disease, all intelligent people must co-operate. The law must support by proper enactments, and these must be enforced with justice and intelligence; it must recognize that the right to enjoy health is quite as sacred as that to possess property; that to poison men in factories and mines, to pollute drinking water-supplies, to adulterate foods and to drug with nostrums is manslaughter. Religion must teach the sanctity of the body as well as that of the soul, that ignorance is sin and knowledge virtue, that parenthood is the holiest function performed by man and that to transmit disease is an unpardonable sin. The teacher must know hygiene as well as mathematics. The capitalist must recognize that improvement in health and growth in intelligence increase the efficiency of labor. There never has been a time when scientific medicine has had so many and such efficient and appreciative helpers as it has to-day. Our sanitary laws are for the most part good, but their administration is weak, on account of ignorance. The pulpits of the land are open, for the most part, to the sanitarian. The respectable newspapers are most effective in the crusade against quackery and disease. The philanthropist has learned that the advancement of science confers the greatest and most lasting benefits on man.

There is a moral obligation to be intelligent. Ignorance is a vice and when it results in injury to any one it becomes a crime, a moral, if not a statutory one. To infect another with disease, either directly or indirectly, as a result of ignorance, is an immoral act. The purpose of government is to protect its citizens, and a government which fails to shelter its citizens against infection is neither intelligent nor moral. To transmit disease of body or mind to offspring is an unpardonable sin. In a reasonable sense it is worse than murder, because it projects suffering into the future indefinitely.

In the further improvement of the physical, mental and moral conditions of the race, medicine should continue to be a leader. There is no other calling so essential to this movement, and in order to more thoroughly fit itself for this important task the profession should first of all look to its own betterment. The medical man should possess intelligence of high order, manifest industry without stint and show the highest integrity in all he does. That is the aim of this Association to attract to its colors men possessing these qualifications and to deny admission to others is shown by the advance in the standard of medical education, the enforcement of medical registration laws and the denunciation of every form of medical charlatanism. In all these directions the profession has the support of the more intelligent men in other callings. The improvement in medical training secured within recent years in this country is without a parallel in the history of education. The requirements for admission to the medical schools have been rapidly advanced and standardized; the number of medical schools has been reduced from 166 to 104 by obliteration and combination, much to the improvement of all, and a far better class of matriculates has been secured. The courses of instruction have been lengthened and made more scientific. Each good medical school is doing more or less of research which is not confined to laboratory investigators, but is fast finding its way into hospitals. Indeed, some of our clinical men are now making most valuable contributions. Every medical man should have much of the spirit of research. It is the pabulum on which medicine feeds and without it the profession atrophies and starves. It is the glory and strength of the profession that it is not bound by dogma and pays no heed to ipse dixits. I have no sympathy with the idea that medical research should be largely relegated to special non-teaching institutions. These have their function and we rejoice in their foundation and support and hope that they may multiply, but the man who is devoid of the spirit of scientific investigation has no place in medicine as student, practitioner or teacher, and the most elaborate medical training without opportunity for scientific observation is barren. Besides, opportunities for medical discovery should be widely distributed. Science makes no provision for an aristocracy. There can be no papal bulls issued in the domain of medicine. The workers must be many, all must be free to pursue knowledge in their own way, and all must be compelled to prove their claims, for "life is short, art is long, opportunity is fleeting, experiment fallacious and judgment difficult."

In this work of self-improvement the profession has had the aid of the more intelligent law makers and administrators. In carrying out these progressive changes there has been much sacrifice of money and personal pride by many members of the profession. Large schools have willingly submitted to marked reduction in the numbers of their students and consequently in financial support. A medical education costs more in time and money than that demanded by any other profession, and the emoluments of the average practitioner have decreased as preventive medicine has become more effective. No other profession pays so heavily the great cost of eradicating the infectious diseases, but this is the function of medicine and no sacrifice should be regarded as too great. While intelligent medical men have been leading the crusade against greed, ignorance and disease, our legislative halls have been crowded with the representatives of sects, cults and charlatans demanding legal recognition. If I mistake not, herculean efforts will be made in the near future to lower the standards demanded of the medical practitioner. These endeavors have been promised aid from those who have heavy financial backing, but if we are worthy of the trust which we bear, we will not yield. We must appeal to the good sense of the people for whose welfare we labor. We must show what scientific medicine has done for the public good and point out the greater things it may do with increased opportunity. It must be admitted that in the crusade for the restriction of tuberculosis many physicians have manifested but little interest. This is shown by their slowness to employ methods of early diagnosis and consequently by their failure to recognize the disease in its curable stage, also by their unwillingness to comply with the laws of notification. It is an undeniable fact that there are many medical men who know less about hygienic measures than the more intelligent of the laity. With advancing knowledge among the masses these professional fossils will be correctly labeled and properly shelved in the local museums of antiquities.

Is the medical profession of this country prepared to do this work? I believe that many of the recent graduates of our best schools are fitted for this highly important function. They may need special training in the courses in public health now being inaugurated. If I mistake not, our profession will soon have wide opportunity to demonstrate its usefulness in this direction. If the public makes this demand, preventive medicine will have the opportunity to do a patriotic service which has never come to any profession at any time. With proper facilities and helpers, health commissioners might within a few years become acquainted with the conditions surrounding every permanent resident within his jurisdiction, and with properly qualified administrators of the law much might be done to abate disease, improve health, increase efficiency, eradicate the venereal diseases, stamp out vagrancy, pauperism, prostitution, alcoholism and crime. Crime is a disease due to heredity or environment, one or both. We now permit it to breed and multiply in our midst. Its causes must be determined and eliminated and its habitations must be discovered, disinfected or destroyed. We have heard too much about the rights of the individual; let us know more about his duties. Too much stress has been laid on the sacredness of private property and too little on the duty of all to contribute to the welfare of the whole. Preventive medicine has demonstrated in a practical way the force of the biblical statements that no man liveth to himself alone, and that every man is his brother's keeper. Preventive medicine is the most potent factor in the socialistic movement of the day with which every good man feels himself more or less in sympathy; besides it is at the same time the most powerful weapon against the anarchy with which some would threaten us.

If preventive medicine is to bestow on man its richest service, the time must come when every citizen will submit himself to a thorough medical examination once a year or oftener. The benefits which would result from such a service are so evident to medical men that detail is not desirable. When recognized in their early stages most of the diseases which now prevail are amenable to treatment. The early recognition of tuberculosis, cancer, diabetes, nephritis, heart disease, etc., with the elimination of the more acute infectious diseases would add something like fifteen years to the average life, besides saving much in invalidism and suffering. The ultimate goal of science is the domination of the forces of nature and their utilization in promoting the welfare of mankind. Science must discover the facts and medicine must make the application for either cure or prevention.—*Journal A. M. A.*

